# Combination NIPS/TIPS Synthesis of α-Fe_2_O_3_ and α/γ-Fe_2_O_3_ Doped PVDF Composite for Efficient Piezocatalytic Degradation of Rhodamine B

**DOI:** 10.3390/molecules28196932

**Published:** 2023-10-04

**Authors:** Asiyat G. Magomedova, Alina A. Rabadanova, Abdulatip O. Shuaibov, Daud A. Selimov, Dinara S. Sobola, Kamil Sh. Rabadanov, Kamal M. Giraev, Farid F. Orudzhev

**Affiliations:** 1 Smart Materials Laboratory, Department of Inorganic Chemistry and Chemical Ecology, Dagestan State University, St. M. Gadjieva 43-a, Dagestan Republic, 367015 Makhachkala, Russia; asiyat_magomedova1996@mail.ru (A.G.M.); rabadanova.alinka@mail.ru (A.A.R.); abdulatip37@mail.ru (A.O.S.); daud-selimov@live.com (D.A.S.); kamal_giraev@mail.ru (K.M.G.); 2 Department of Physics, Faculty of Electrical Engineering and Communication, Brno University of Technology, Technicka 10, 616 00 Brno, Czech Republic; 3 Amirkhanov Institute of Physics of Dagestan Federal Research Center, Russian Academy of Sciences, 367003 Makhachkala, Russia; rksh83@mail.ru

**Keywords:** polyvinylidene fluoride, PVDF, α-Fe_2_O_3_, γ-Fe_2_O_3_, piezocatalysis, piezopotential, Rhodamine B, NIPS, TIPS, membrane

## Abstract

Highly porous membranes based on polyvinylidene fluoride (PVDF) with the addition of nanoscale particles of non-magnetic and magnetic iron oxides were synthesized using a combined method of non-solvent induced phase separation (NIPS) and thermo-induced phase separation (TIPS) based on the technique developed by Dr. Blade. The obtained membranes were characterized using SEM, EDS, XRD, IR, diffuse reflectance spectroscopy, and fluorescent microscopy. It was shown that the membranes possessed a high fraction of electroactive phase, which increased up to a maximum of 96% with the addition of 2 wt% of α-Fe_2_O_3_ and α/γ-Fe_2_O_3_ nanoparticles. It was demonstrated that doping PVDF with nanoparticles contributed to the reduction of pore size in the membrane. All membranes exhibited piezocatalytic activity in the degradation of Rhodamine B. The degree of degradation increased from 69% when using pure PVDF membrane to 90% when using the composite membrane. The nature of the additive did not affect the piezocatalytic activity. It was determined that the main reactive species responsible for the degradation of Rhodamine B were ^•^OH and ^•^O_2_^−^. It was also shown that under piezocatalytic conditions, composite membranes generated a piezopotential of approximately 2.5 V.

## 1. Introduction

With the rapid development of global industrialization, the amount of non-degradable oils and soluble dyes in the natural water cycle is increasing, posing a significant threat to ecosystem stability and human health [1]. Water scarcity, along with an increasing water demand, is one of the greatest global problems of the century. At the same time, the discharge of hazardous compounds into water from urban and industrial sectors remains a problem due to the presence of many chemicals in various concentrations. It is also becoming evident that modern wastewater treatment technologies are reaching their limits [2]. Research continues to confirm that advanced oxidation processes (AOPs) are the most promising and competitive innovative methods for water and wastewater treatment to remove biologically resistant compounds. AOPs are based on a physicochemical process that generates chemically active compounds at ambient temperature and pressure, with or without a catalyst, subsequently converting organic pollutants into carbon dioxide and water [3].

Piezoelectric materials have promising potential for converting mechanical energy into chemical energy by combining the piezotronic effect with electrochemical processes. The piezopotential generated by an external force can effectively separate free carriers (electrons and holes), allowing piezoelectric materials to be directly used for renewable energy production and environmental restoration using weak mechanical forces such as noise and vibration from the surroundings [4].

The piezocatalytic efficiency in organic pollutant removal has been demonstrated in studies [5,6,7,8]. From these works, it can be concluded that piezocatalysis is a promising technology for the wastewater treatment of organic pollutants on an industrial scale. However, dispersed heterogeneous piezocatalysts have several common key disadvantages, such as fragility, high cost, complexity of manufacturing, and being sources of secondary pollution. Against this background, organic piezocatalysts appear much more promising, among which polyvinylidene fluoride (PVDF) and its copolymers from the family of fluorinated ethylene polymers stand out. Their advantages include environmental friendliness, biocompatibility, flexibility, and high resistance to halogens and acids [9].

The polymer possesses strong piezoelectric, magnetostrictive, and pyroelectric properties [10]. PVDF is a semi-crystalline polymer with up to five polymorphic modifications: α, β, γ, δ, and ε. The presence of piezoelectric properties is explained by the polar β-TGTG’ (trans-gauche-trans-gauche’) and partially polar γ-T3GT3G’ (trans-trans-trans-gauche-trans-trans-trans-gauche’) phases, with the β-phase contributing the most to the piezoelectric properties due to its significantly stronger dipole moment [11]. Therefore, to enhance the efficiency of PVDF-based piezocatalysts, increasing the proportion of the β-phase is necessary. It has been demonstrated that alignment of dipoles and an increase in the beta polymorph can be achieved through various methods such as stretching [12], polarization in electric fields during synthesis [13,14], thermal annealing [15], introduction of fillers [16], etc. However, the method of membrane synthesis also has a strong influence on the polymer structure. One popular and cost-effective method for synthesizing polymer membranes is non-solvent-induced phase separation (NIPS) [17]. NIPS membranes have a dense surface with non-uniform morphology [18], suitable for catalytic applications as active centers in redox processes. For example, in a study [19], the aging of PVDF membranes manufactured using NIPS and thermal phase separation (TIPS) was investigated, revealing that the dominant phase in the NIPS-PVDF membrane was β-phase, while the TIPS-PVDF membrane consisted mainly of α-phase.

In recent years, there has been a significant increase in interest in the study of piezoelectric polymer composites based on PVDF for water purification purposes [20]. Various functional materials, such as PVDF-HFP@rGO [21], PVDF/BaTiO_3_ [22], PVDF/MoS_2_ [23], and PVDF/Cu_3_B_2_O_6_ [24], have been used as fillers for the PVDF matrix. For instance, in a study [25], Fe_3_O_4_/PVDF membranes were synthesized using non-solvent-induced phase inversion and utilized for Fenton catalytic oxidation of Methylene Blue. The results showed that the membrane exhibited good hydrophilicity, thermal stability, and excellent catalytic activity with a 97% degradation efficiency of the pollutant. In another study [22], the piezocatalytic activity of a hydrophilic porous BaTiO3/PVDF membrane was investigated for the decomposition of BPA (20 mL, 15 mg/L), using an ultrasonic bath (40 kHz, 300 W) as a source of mechanical stress. The catalytic degradation efficiency of BPA reached 95% within 60 min.

Fe_2_O_3_ is widely used as a potential catalyst in various advanced oxidation processes (AOPs) for the degradation of dyes and organic pollutants. Furthermore, the magnetic properties of Fe_2_O_3_ have attracted significant interest in creating composites with other materials to facilitate the catalyst recovery and reuse process. For example, it has been shown that maghemite exhibits low sonocatalytic activity in the degradation of Orange G dye [26]. However, the modification of maghemite by creating an interfacial boundary with TiO_2_ significantly enhances its catalytic activity.

Despite the significant progress in using Fe_2_O_3_ in recent years, its efficiency as a sole catalyst is still unsatisfactory. This is mainly due to inefficient charge migration and transfer, which arise from several drawbacks of Fe_2_O_3_, such as short hole diffusion distance (2–4 nm), fast charge recombination (6 ps), and poor carrier migration. Therefore, the development of efficient Fe_2_O_3_-based catalysts with outstanding catalytic characteristics remains a challenging task for researchers.

In this study, we synthesized a flexible porous PVDF membrane using the NIPS/TIPS method and investigated the effect of doping it with α/γ-Fe_2_O_3_ and α-Fe_2_O_3_ nanoparticles. We examined the piezocatalytic activity of the resulting composites in the decomposition of a model dye, Rhodamine B.

## 2. Experimental

### 2.1. Synthesis of α/γ-Fe_2_O_3_ and α-Fe_2_O_3_ Particles

The synthesis of α/γ and α-Fe_2_O_3_ was carried out using the combustion method of glycine-nitrate precursors [27,28,29]. An aqueous iron (III) nitrate solution was used as the starting material, and glycine was used as the fuel. The precursor was prepared by mixing glycine and Fe(NO_3_)_3_ in an aqueous solution. The resulting solution was evaporated to form a homogeneous gel on an electron heater with a working temperature of approximately 180 °C. Upon further heating, the reaction mixture ignited and formed iron (III) oxide powder. The combustion was fast and self-sustaining, with a flame temperature ranging from 1100 to 1450 °C. The synthesized samples were annealed at 400 °C for 1 h.

### 2.2. Synthesis of PVDF Membrane

The membranes were fabricated using a Dr Blade method, combining non-solvent-induced phase separation (NIPS) and thermally induced phase separation (TIPS) methods. A PVDF polymer solution (Galopolymer, Perm, Russia) with a mass concentration of approximately 20% dimethyl sulfoxide was stirred on a vortex shaker for 15 min until homogeneous consistency was achieved. The solution was then placed in an ultrasonic bath at 50 °C for complete homogenization and degassing (30 min). The solution was poured onto glass, and a film was obtained using a Dr Blade with a gap of 100 μm. After that, the glass with the film was transferred to a container with distilled water. After 40 min, the prepared membrane was transferred onto aluminum foil and placed in a drying oven, where it was kept at a temperature of 80 °C for 24 h.

**Figure 1:**
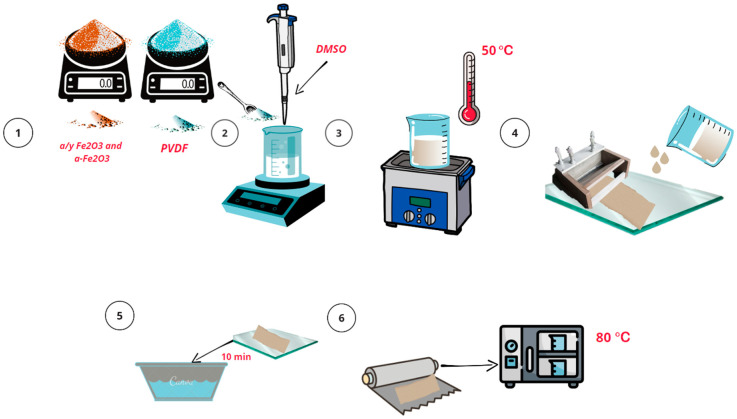
Schematic representation of the membrane synthesis procedure.

A similar procedure was followed for the synthesis of composite α-Fe_2_O_3_ and α/γ-Fe_2_O_3_ doped PVDF membranes, with the addition of 2 wt% corresponding nanoparticles to the polymer solution in stage 2. The subsequent process was carried out under conditions identical to those described above. The schematic representation of the synthesis procedure is shown in Figure 1.

### 2.3. Methods

The morphology of synthesized samples was investigated using scanning electron microscopy (SEM) (ASPEX Express, Delmont, PA, USA) with an EDX attachment.

Infrared spectra of polymer composites based on PVDF were recorded on a Fourier-transform infrared (FTIR) spectrometer (Vertex 40, Bruker, Germany) in the range of 400–4000 cm^−1^ with a resolution of 2 cm^−1^ in absorbance mode.

The crystalline phase of the samples was determined by X-ray diffraction patterns obtained from an Empyrean PANalytical X-ray diffractometer with CuK α radiation (λ = 1.5418 Å).

For measuring the optical properties, the samples were placed between two quartz plates with a refractive index of ${\bar{n}}_{gl}=1.55$. The samples themselves were thin films measuring 10.0 × 10.0 mm and with a thickness of d = 100.0 ± 10.0 μm, and their refractive index was assumed to be ${\bar{n}}_{p}=1.45$. Since PVDF samples exhibit a high level of light scattering, as previously shown in [13,16], measurements of both transmittance spectra ($T_{t}$) and reflectance spectra ($R_{d}$) were necessary to obtain accurate spectrophotometric data. In this study, these measurements were performed using an integrating sphere Avasphere-50 (Avantes, Apeldoorn, Nederland) in the wavelength range of λ~300–1000 nm. The combined deuterium/halogen lamp AvaLight-DH-S-BAL (Avantes, Apeldoorn, Nederland) was used as the light source, and its emission was delivered to the samples via a 600 μm fiber optic light guide. Photonic signals were collected using a bifurcated 600 μm light guide and recorded with an MS3504i spectrometer (SOL-Instruments, Minsk, Belarus) combined with an HS-101(HR)-2048 × 122 CCD camera (Hamamatsu, Hamamatsu City, Japan) and a personal computer. Three series of measurements were performed for each sample. The result for the absorption and scattering spectra was determined by averaging the series of measurements using the formula:

(1) $$\delta =\sqrt{\frac{\sum_{i=1}^{n}{\left(\bar{\sigma }-\sigma_{i}\right)}^{2}}{n\left(n-1\right)}}$$

where n—is the number of series measurements, $\sigma_{i}$—is the values of the coefficients $\mu_{a}$ и $\mu_{s}^{{}^{\prime }}$ for the i-th sample, and $\bar{\sigma }$—is the average value of the optical coefficients, calculated as—$\sum_{i=1}^{n}\frac{\sigma_{i}}{n}$.

Microfluorescence properties were investigated using a laser scanning confocal microscope MR-350 (SOL-Instruments, Minsk, Belarus) equipped with a Nikon-NiU optical microscope (Nikon, Tokyo, Japan). The fluorescence signals were excited using laser radiation at a wavelength of $\lambda_{ex}=408\;\mathrm{nm}$. Analysis of the photoluminescence signals was also performed using an automated MS3504i spectrometer in the wavelength range of λ~420–800 nm.

### 2.4. Piezocatalytic Experiment Methodology

As the main model organic substance, a solution of Rhodamine B (Rh B) with a concentration of 8 mg/L was used. Samples of PVDF polymer membrane, α,γ-Fe_2_O_3_/PVDF, and α-Fe_2_O_3_/PVDF with dimensions of 3 × 1 cm were immersed in a glass containing 20 mL of Rh B solution. It should be noted that due to the hydrophobicity of the membrane, it always remained on the solution’s surface. An ultrasound bath (FanYing Sonic, 2L, Shenzhen, China) with a power of 120 W and a frequency of 40 kHz was used as a source of mechanical stimulation (to induce the piezoelectric effect). The position of the glass in the ultrasound bath and its immersion depth were fixed at the center for all experiments. The bath maintained a constant temperature of 26 °C. The experiment lasted for 60 min, with samples taken every 15 min. The sample was analyzed using a spectrophotometer SF-2000 at a wavelength of 553.7 nm. The following experiments were conducted to decompose Rh B under ultrasound without a catalyst (sonochemical degradation) to differentiate the effects of different factors. The degree of dye decomposition was determined by the ratio of the current dye concentration to the initial concentration.

For the analysis of ^•^OH fluorescence, the experiment was conducted under similar piezocatalytic conditions. Instead of Rh B solution, 3 mmol terephthalic acid was used, and the pH was adjusted to 10.0 using NaOH solution. The fluorescence spectra of the solution were recorded using a Hitachi F-4500 fluorescence spectrophotometer with an optical path length of 1.0 cm, excited at a wavelength of 310 nm, and the fluorescence emission peak at 430 nm was recorded at fixed time intervals.

The generation of ^•^O_2_^−^ was also investigated using a similar piezocatalytic procedure. A solution of Nitro blue tetrazolium chloride (NBT) with a concentration of 20 mg/L was used as the test solution, and approximately 4 mL of the NBT solution was taken at specific time intervals for analysis using spectrophotometry to determine the optical density at 259 nm.

### 2.5. Piezoelectric Nanogenerator Measurement

To fabricate the piezoelectric nanogenerator (PNG), a membrane with an area of 3 × 1 cm^2^ was placed between two aluminum foil electrodes. The entire multilayer structure was then carefully laminated with polypropylene (PP) adhesive tape for compactness. The measurement of piezopotential was performed using an ultrasound bath with a power of 120 W and a frequency of 40 kHz (FanYing Sonic, 2L, China). The sample was immersed in the filled ultrasound bath, securely fixed in place, and the ultrasound was cyclically turned on and off. A programmable Arduino UNO R3 analog-to-digital converter (ADC) in Arduino 1.8.19 application was used to measure the output voltage. A 2 MΩ resistor was also used in the circuit to eliminate unwanted noise.

## 3. Results and Discussion

**Figure 2:**
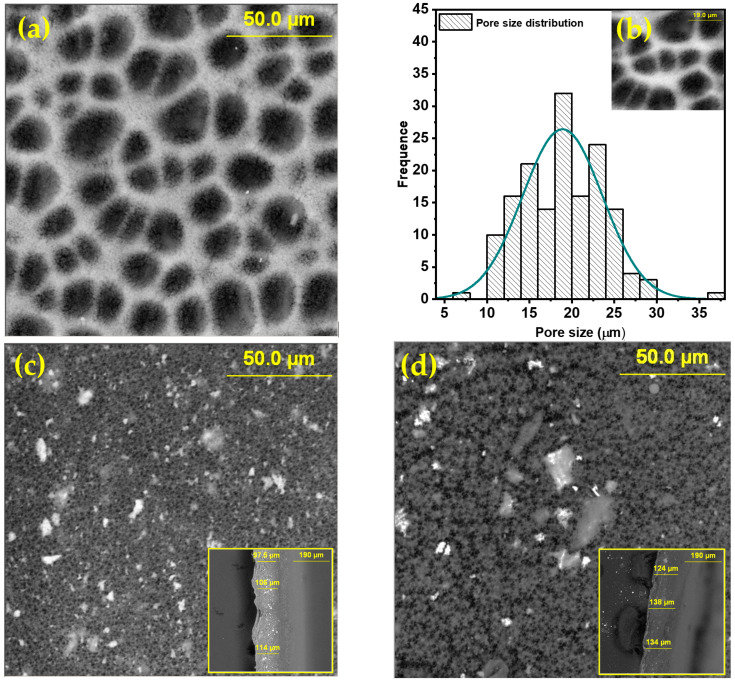
SEM morphology of membranes: (**a**) PVDF; (**c**) α-Fe_2_O_3_/PVDF; (**d**) α,γ-Fe_2_O_3_/PVDF; (Inserts: cross-sectional images); (**b**) size distribution of pores in pure PVDF.

Scanning electron microscopy (SEM) with energy-dispersive spectroscopy (EDS) was used for the comprehensive analysis of the morphology and chemical composition of the membranes. SEM images were analyzed using ImageJ software to evaluate the size distribution of pores. Figure 2 shows SEM images of pure and doped membranes.

In the presented images, it can be observed that the upper part of the pure PVDF membrane exhibits a homogeneous and highly porous cellular structure. The distribution and sizes of pores are uneven. It was determined that the pore diameter and interval are approximately 20 μm and 1.5 μm, respectively. SEM analysis from the bottom side of the membrane revealed that the lower surface is also porous.

Although many studies have reported strong asymmetry in pore distribution between the top and bottom surfaces [30], in our case, the same structure is achieved by practically instant separation of the membrane from the substrate during immersion in a non-solvent with phase inversion. SEM analysis of composite membranes (Figure 2c,d) showed that the surface is denser and does not contain submicron pores. The bright areas in the images of composite membranes represent nanoparticles uniformly distributed throughout the PVDF matrix. The inclusion of nanoparticles in the PVDF matrix leads to a significant reduction in pore size. For composite membranes, pore sizes are already in the nanometer range. From the SEM images of cross-sectional cuts in the insets of Figure 2c,d, it is evident that the membrane thickness is approximately 100–120 μm, and the structure is symmetrical throughout the cross-section. The EDX spectra of the membranes, obtained from the area shown in the SEM images in Figure 2, are presented in Figure S1 in the Supplementary File [20,23,24,31,32,33,34,35]. It can be seen that the samples are chemically pure and free from impurities. The presence of the Al peak is associated with the substrate used to mount the samples in the chamber.

**Figure 3:**
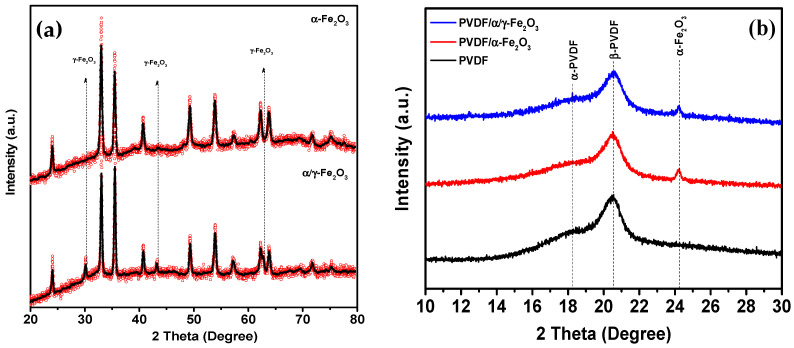
(**a**) X-ray diffraction patterns of pure nanoparticles of α-Fe_2_O_3_ and α,γ-Fe_2_O_3_; (**b**) X-ray diffraction patterns of PVDF membrane, α-Fe_2_O_3_/PVDF, and α,γ-Fe_2_O_3_/PVDF.

Since it is known that PVDF is a semi-crystalline polymer, it is necessary to determine whether the synthesized materials possess a crystalline structure. For this purpose, structural analysis was performed using X-ray diffraction. The spectrum is shown in Figure 3a.

Figure 3a shows X-ray diffraction patterns of pure α-Fe_2_O_3_ and α,γ-Fe_2_O_3_ nanoparticles. All observed peaks can be indexed according to the rhombohedral (hexagonal) structure of α-Fe_2_O_3_ (space group: R-3c) with lattice parameters a = 0.5034 nm and c = 1.375 nm. The spectrum is characterized by peaks at 2θ angles of 24.16°, 33.12°, 35.63°, 40.64°, 49.47°, 54.08°, and 57.42°, corresponding to diffraction planes 012, 104, 110, 113, 024, 116, and 018. The narrow, sharp peaks indicate that the sample is highly crystalline. Using the Scherrer equation, the crystallite size of α-Fe_2_O_3_ was determined to be 63.9 nm. The XRD spectrum of the mixed-phase sample is well described by two phases: α-Fe_2_O_3_ with a hexagonal structure and space group R-3c (Ref. Code 98-006-6756), and γ-Fe_2_O_3_ with a cubic structure and space group Fd-3m (Ref. Code 98-024-7036). Quantitative phase analysis using the Rietveld method showed that α-Fe_2_O_3_ exists as the main phase (79.6%), while γ-Fe_2_O_3_ is present in a quantity of 20.4% (see Figure S2 Supplementary File) [20,23,24,31,32,33,34,35]. The average crystallite size of the α-phase was found to be 47.4 nm, while that of the γ-phase was 45.7 nm [29].

**Figure 4:**
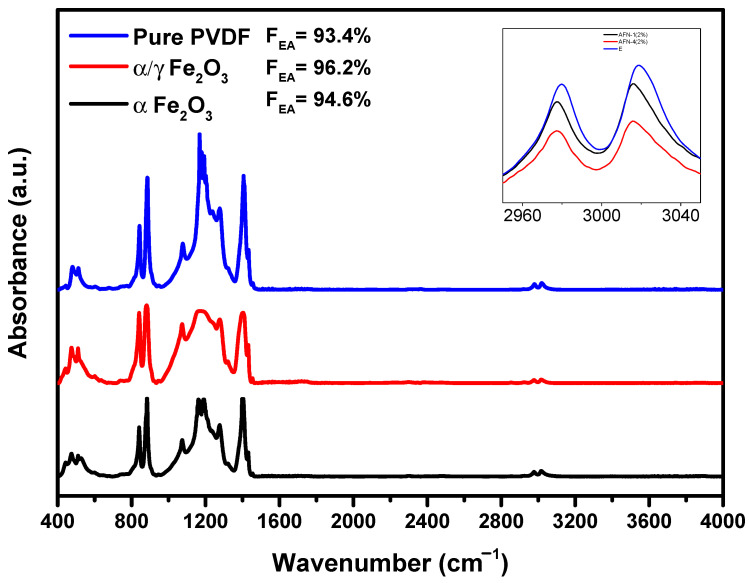
FTIR absorption spectra of PVDF, α-Fe_2_O_3_/PVDF, and α,γ-Fe_2_O_3_/PVDF membranes.

Figure 3b shows X-ray diffraction patterns of PVDF, α-Fe_2_O_3_/PVDF, and α/γ-Fe_2_O_3_/PVDF membranes, where the main peaks of PVDF are visible [36]. At diffraction angles of 18.4° and 20°, two intense peaks appear, corresponding to diffraction planes α (002) and β (110), respectively. A broad shoulder around 18.8° could correspond to either the (002) plane of the γ-phase of PVDF or the (110) plane of the monoclinic α-phase. Although XRD indicates that the predominant phase of the polymer in all membranes is the electroactive β and γ phases, an accurate quantitative assessment of the ratio between polar and nonpolar phases is challenging. To address this issue, FTIR spectra were obtained. Figure 4 shows the FTIR spectra of PVDF, α-Fe_2_O_3_/PVDF, and α/γ-Fe_2_O_3_/PVDF membranes.

The absorption bands observed at 976, 796, 764, and 611 cm^−1^ correspond to the nonpolar α-phase of PVDF, while the characteristic peaks at 1276, 1233, and 837 cm^−1^ correspond to the electroactive β- and γ-phases. [37,38]. The intensity of all characteristic absorption bands representing the nonpolar α-phase decreases in the case of composite membranes compared to pure PVDF. Additionally, the intensity of the characteristic bands for both β- and γ-phases of PVDF was increased for the composites. This is due to interphase interactions between the fillers and the dipoles -CH_2_-/–CF_2_- chains of PVDF, leading to the formation of electroactive phases [39,40].

The infrared Fourier spectra in the range of 3600–2800 cm^−1^ shown on the tab of Figure 4 indicate the formation of intermolecular bonds between the PVDF molecular chain and the filler nanoparticles [41]. The two main vibrational bands of -CH_2_- for asymmetric (ν_as_) and symmetric (ν_s_) stretching vibrations are shifted to the low-frequency region due to interphase interactions between the positively charged surface of Fe_2_O_3_ filler and the -CF_2_- dipoles of the PVDF matrix.

The relative content of the electroactive phase (sum of β- and γ-phases) in pure and composite PVDF membranes was calculated using the following equation:

(2) $$F_{EA}=\frac{I_{EA}}{\left(\frac{K_{840^{*}}}{K_{763}}\right)I_{763}+I_{EA}}\times 100\%$$

where, *I*_EA_ and *I*_763_ are the absorbencies at 840* and 763 cm^−1^, respectively; *K*_840*_ and *K*_763_ are the absorption coefficients at the respective wave numbers, whose values are 7.7 × 10^4^ and 6.1 × 10^4^ cm^2^ mol^−1^, respectively [38]. 

According to the equation, the content of the electroactive phase in PVDF and its composites was estimated. It was found that the relative fraction of the electroactive phase is higher for PVDF nanocomposites than pure PVDF. These results can be explained by the smaller size of the nanocrystals and the stabilization of the phase due to the positive charges present in the nanoparticles, which interact with the -CF_2_- dipoles of the PVDF chains.

**Figure 5:**
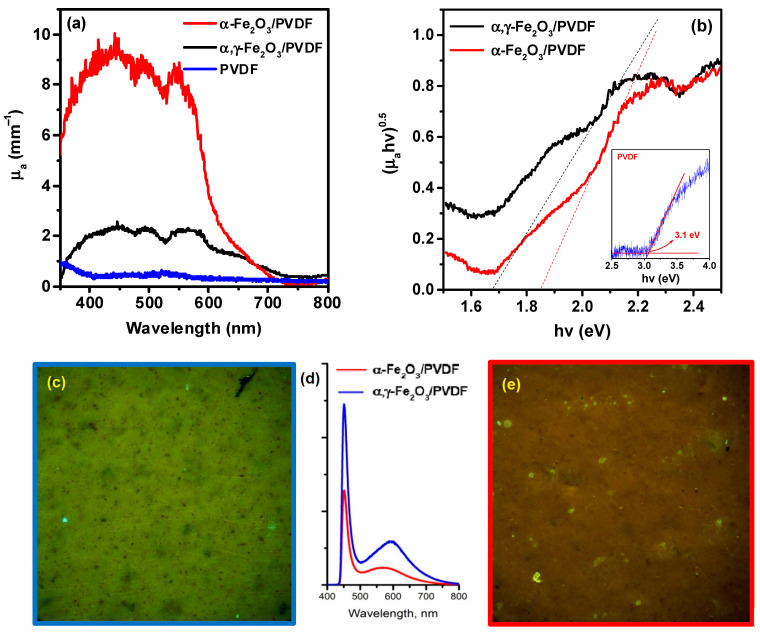
Spectra of the optical absorption coefficient μ_a_ (**a**) and Tauc plots (**b**). Fluorescent micrographs of α,γ-Fe_2_O_3_/PVDF (**c**) and α-Fe_2_O_3_/PVDF (**e**) at 500× magnification, as well as photoluminescence spectra (**d**).

The optical properties of the membranes are especially important, as semiconductor nanoparticles are used as fillers, and one of the possible mechanisms of ultrasound impact on the system is cavitation sonoluminescence, which can activate a photocatalytic mechanism. Therefore, the membranes were investigated using diffuse reflection electron spectroscopy, photoluminescence spectroscopy, and fluorescence microscopy. The data are presented in Figure 5. 

The final data for the spectrophotometric coefficients of total transmittance $T_{t}$ and diffuse reflection $R_{d}$ were determined as:

(3) $$R_{d}^{exp}=\frac{R_{d}^{s}\left(\lambda \right)-R_{0}\left(\lambda \right)}{R_{gl}\left(\lambda \right)-R_{0}\left(\lambda \right)}$$

(4) $$T_{t}^{exp}=\frac{T_{t}^{s}\left(\lambda \right)-T_{0}\left(\lambda \right)}{T_{gl}\left(\lambda \right)-T_{0}\left(\lambda \right)}$$

where $T_{t}^{s}\left(\lambda \right)$ and $R_{d}^{s}\left(\lambda \right)$—are the transmission and reflection spectra of the samples; $T_{gl}\left(\lambda \right)$ and $R_{gl}\left(\lambda \right)$—are the spectra of the reference signal measured with quartz plates; $T_{0}\left(\lambda \right)$—is the signal of the integrating sphere with the input port covered and the output port open; $R_{0}\left(\lambda \right)$—is the signal for the sphere with the optical ports open.

To calculate the spectral dependence of the optical absorption coefficient–$\mu_{a}$ and light scattering–$\mu_{s}^{{}^{\prime }}$ the inverse method of Monte Carlo numerical modeling was used, the algorithm of which can be briefly represented by the sequential implementation of the following steps:

1. Calculation of initial approximations for the coefficients $\mu_{a}$ and $\mu_{s}^{{}^{\prime }}$ based on the $T_{t}^{exp}$ and $R_{d}^{exp}$ data, using the relationships:
  (5) $$\frac{\mu_{s}^{{}^{\prime }}}{\mu_{a}+\mu_{s}^{{}^{\prime }}}=\left\{\begin{array}{c} 1-{\left(\frac{1-4R_{d}^{exp}-T_{t}^{exp}}{1-T_{t}^{exp}}\right)}^{2},\;at\;\frac{R_{d}^{exp}}{1-T_{d}^{exp}}<0.1\\ 1-{\frac{4}{9}\left(\frac{1-R_{d}^{exp}-T_{t}^{exp}}{1-T_{t}^{exp}}\right)}^{2},\;at\;\frac{R_{d}^{exp}}{1-T_{d}^{exp}}\geq 0.1 \end{array}\right.$$
  (6) $$\left(\mu_{a}+\mu_{s}^{{}^{\prime }}\right)d=\left\{\begin{array}{c} -\frac{lnT_{t}^{exp}ln0.05}{lnR_{d}^{exp}},\;at\;R_{d}^{exp}\leq 0.1\\ 2^{1+5\left(R_{d}^{exp}-T_{t}^{exp}\right)},\;at\;R_{d}^{exp}>0.1 \end{array}\right.$$
  where *d* is the thickness of the samples.
2. Calculation of the parameters $T_{t}^{exp}$ and $R_{d}^{exp}$ based on the obtained values of the coefficients $\mu_{a}$ and ${\mu {}^{\prime }}_{s}$, using the Monte Carlo method.
3. Construction of the objective function and its minimization:
  (7) $$F={\left(T_{t}^{exp}-T_{t}^{calc}\right)}^{2}+{\left(R_{d}^{exp}-R_{d}^{calc}\right)}^{2}$$
4. The minimization procedure was performed based on the Nelder-Mead simplex method until the condition is met:
  (8) $$\frac{\left|T_{t}^{exp}-T_{t}^{calc}\right|}{T_{t}^{exp}}+\frac{\left|R_{d}^{exp}-R_{d}^{calc}\right|}{R_{d}^{exp}}\leq 0.01$$

Typical spectra of the absorption coefficient $\mu_{a}$ for PVDF samples, averaged over measurement series, in terms of wavelength and energy scales are shown in Figure 5a and Figure 5b, respectively.

As seen from Figure 5a, the $\mu_{a}$ coefficient of the composite membranes represents a spectral contour formed by wide overlapping bands of intense absorption with peaks in the wavelength ranges of 435.0 ± 10.0, 495.0 ± 10.0, and 550.0 ± 10.0 nm. Considering light scattering on the elements of the morphological structure of the polymer films allowed determining the correct absorption value, which for α-Fe_2_O_3_/PVDF samples in the extremum range was $\mu_{a}^{435-550}=9.0\pm 0.5$ mm^−1^. The absorption spectrum of pure PVDF is characterized by almost complete absence of absorption in the entire investigated range except for slight absorption in the 300 nm region.

Significant changes in the spectral characteristics of $\mu_{a}$ are observed for the α,γ-Fe_2_O_3_/PVDF membrane: a sharp decrease in the overall level of the absorption coefficient (by a factor of 4) is accompanied by an increase in the intensity of absorption bands in the spectral range of 550–800 nm. This fact is also clearly visible on the energy absorption spectrum (Figure 5b), which shows normalized $\mu_{a}$ spectra. The hypsochromic shift of the long-wavelength bands leads to both an energy shift and a change in the slope of the extrapolation lines. According to the well-known Tauc expression, this indicates a decrease in the values of the bandgap width from 1.88 eV for α-Fe_2_O_3_/PVDF polymer samples to 1.68 eV for α,γ-Fe_2_O_3_/PVDF samples. As can be seen, these values are significantly lower than the bandgap width of pure PVDF, which is 3.1 eV, as shown in the inset of Figure 5b.

The results of the fluorescent microscopy studies are shown in Figure 5c–e. As can be seen, under the excitation wavelength of $\lambda_{ex}=408\;nm,$ and at 500× magnification, the surface of the α,γ-Fe_2_O_3_/PVDF membrane (Figure 5c) appears as a solid homogeneous structure without pronounced morphological features, emitting a greenish-yellow light due to its fluorescence. At the same time, red fluorescent micro-inclusions can be observed in the field of view, uniformly distributed across the micrograph, which presumably correspond to clusters of α,γ-Fe_2_O_3_ nanoparticles. In comparison, for the α-Fe_2_O_3_/PVDF sample (Figure 5e), a more pronounced red emission is characteristic, presumably caused by exclusively α-Fe_2_O_3_ nanoparticles, against which areas of polymer fluorescence can be seen.

The results of microfluorescence studies are also reflected in the spectra of laser-induced photoluminescence of PVDF samples (Figure 5d). Using confocal mode allowed us to identify common patterns and distinctive features in the re-emission spectra. For α,γ-Fe_2_O_3_/PVDF and α-Fe_2_O_3_/PVDF, fluorescence spectra are formed by at least two emission bands-an intense component at a wavelength of $\lambda_{em}\;\sim 455.0\pm 5.0$ nm and less intense broadband in the spectral range of $\lambda_{em}\;\sim 560\pm 15.0$ nm. At the same time, for α,γ-Fe2O3/PVDF, a decrease in fluorescence intensity by 1.5 times is observed at the wavelength of the main peak (455.0 ± 5.0 nm), as well as a shift of the maximum of the red-shifted component by 50 ± 10.0 nm towards longer wavelengths. It is important to note that the results of fluorescence studies largely explain the peculiarities of the spatial distribution of fluorescent components and are in good agreement with the data of optical studies.

**Figure 6:**
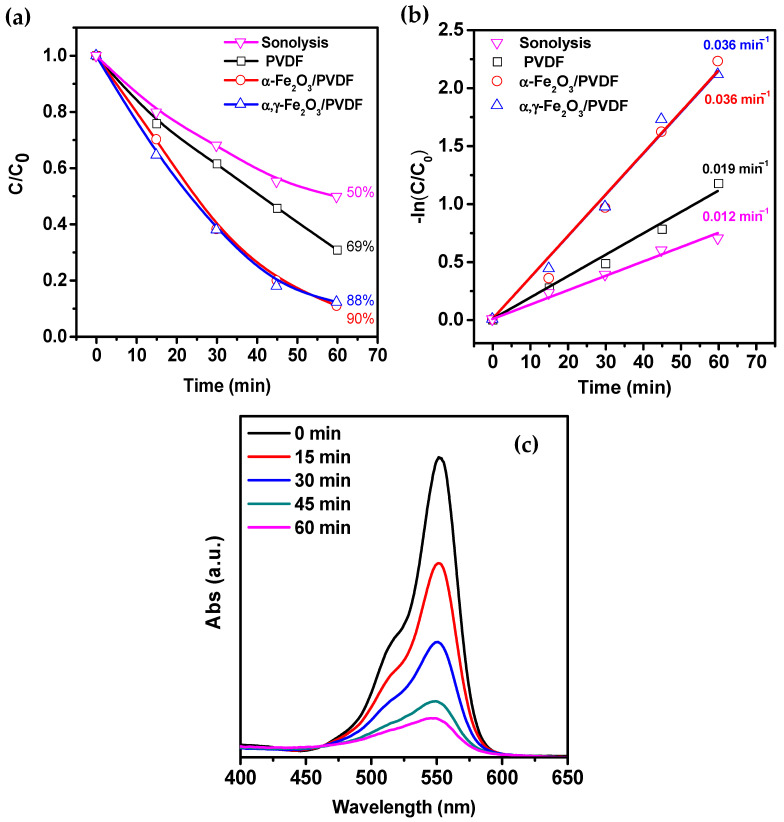
(**a**) Piezocatalytic degradation curves of RhB using different membranes. (**b**) Kinetic curves of the piezocatalytic degradation rates of RhB using different membranes. (**c**) The typical absorption spectrum of RhB during the piezocatalysis process using a composite catalyst.

The piezocatalytic characteristics of the samples were investigated based on their ability to degrade RhB under ultrasound exposure. As shown in Figure 6a, the degradation efficiency of pure PVDF is insignificant compared to sonolysis efficiency. When the solution is subjected to ultrasound treatment without a catalyst, the degradation efficiency reaches 50%. The sonolysis effect is mainly due to the cavitation effect [42]. It has been shown that compounds with high volatility are easily and directly destroyed inside cavitation bubbles (direct sonolysis) [43], while non-volatile compounds decompose through interaction with short-lived radicals formed through cavitation pyrolysis of water.

When pure PVDF is used as the piezocatalyst, the degradation efficiency reaches 69%, indicating that PVDF exhibits piezocatalytic activity. The acceleration of the reaction compared to sonolysis, as seen from the kinetic curves in Figure 6b, is 1.6 times higher and is attributed to the generation of piezopotential in the polymer under ultrasonic (US) irradiation. Composite catalysts show higher catalytic activity, achieving a degradation degree of 90% within 60 min. Furthermore, the reaction rate is three times higher than sonolysis and 1.9 times higher than pure PVDF. 

**Figure 7:**
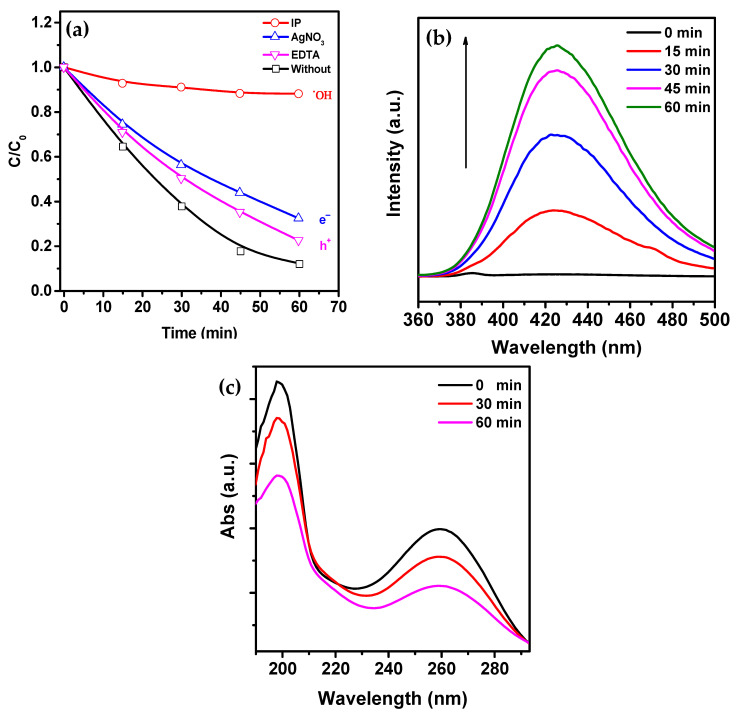
(**a**) RhB piezocatalytic degradation curves for α-Fe_2_O_3_/PVDF in the presence of different absorbers. (**b**) Fluorescence spectra of 2-hydroxyterephthalic acid for ^•^OH detection. (**c**) UV-visible absorption spectra of NBT solution for ^•^O_2_^−^ detection.

To investigate the mechanism of piezocatalysis, trapping experiments were conducted to identify the active oxygen species generated in the α-Fe_2_O_3_/PVDF system. Three different scavengers, AgNO_3_, IPA, and EDTA-2Na, were used to capture e^−^, ^•^OH, and h^+^ species, respectively. As shown in Figure 7a, adding IPA as a ^•^OH scavenger significantly reduced the degradation efficiency to 12%, indicating that ^•^OH species made a dominant contribution to the degradation. On the other hand, adding EDTA-2Na and AgNO_3_ caused a slight decrease in degradation efficiency to 78% and 68%, respectively. This suggests a limited contribution of piezoelectrically induced e^−^/h^+^ species to the degradation.

To detect possible active species during dye degradation, the generation of ^•^OH and ^•^O_2_^−^ was investigated using fluorescent and UV-visible spectra with terephthalic acid and NBT as molecular probes, respectively, as shown in Figure 7b,c. The absence of a distinct peak at the beginning of the experiment (Figure 7b) indicates the absence of ^•^OH in the reaction solution. However, after ultrasound vibration, the characteristic fluorescence peak at a wavelength of 425 nm gradually increases due to the reaction between terephthalic acid molecules and ^•^OH, forming fluorescent 2-hydroxyterephthalic acid. 

In Figure 7c, the characteristic absorption peak at a wavelength of 259 nm continuously decreases during the experiment due to the formation of formazan resulting from the reaction between NBT and ^•^O_2_^−^. Considering that the peak intensity at a wavelength of 259 nm slowly decreases, the generation of ^•^O_2_^−^ cannot be ignored either. Thus, these results confirm that both ^•^OH and ^•^O_2_^−^ are formed during the catalytic reaction.

Based on the obtained results, the mechanism of RhB piezocatalytic degradation can be presented as follows. Piezocatalysis using pure PVDF membrane occurred due to piezoelectricity from the electroactive phase. In these materials, under the action of mechanical stress (in this case, from the pressure exerted by shock waves during cavitation collapse), an increased polarization field is generated. The piezoelectrocatalytic mechanism (PEC) is realized. In the case of composite membranes, piezoelectricity can increase as the proportion of the electroactive phase increases. However, as can be seen, a slight increase in the proportion of the electroactive phase leads to a significant enhancement of piezocatalytic activity. This may say that some other factor handles the enhancement of piezocatalytic activity.

Considering the presence of iron oxide in the membrane matrix [44], there are several other probable piezocatalysis mechanisms besides piezoelectrocatalysis:

1. Photocatalytic mechanism (PCM), in which sonoluminescence is generated during cavitation collapse, can contribute to the formation of electron-hole pairs in iron oxide.
2. Thermocatalytic mechanism (TCM), in which, according to the hot spot theory, the local high temperature during cavitation collapse can promote the formation of electron-hole pairs in iron oxide through thermo-induced thermal excitation of the semiconductor.

It has been previously reported that hematite shows low photocatalytic activity due to the low hole mobility (10^−2^ cm^2^ V^−1^ S^−1^) and very high electron-hole recombination rate (around 6 ps) [45].

**Figure 8:**
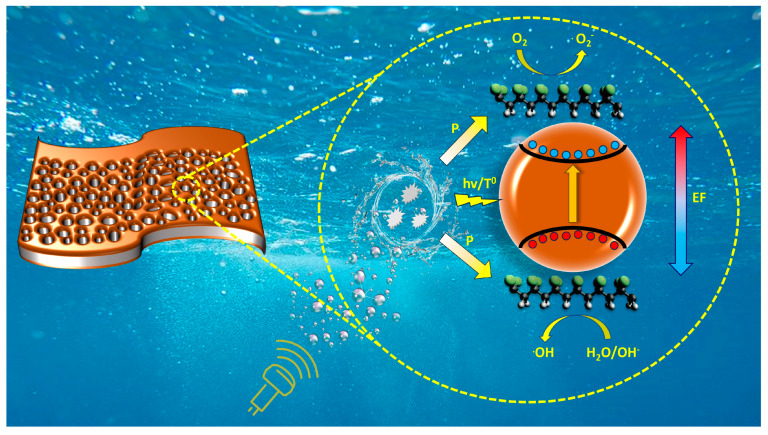
Scheme of piezocatalysis of α-Fe_2_O_3_/PVDF composite film.

However, in this case, PCM and TCM should not be neglected, as the bending of bands caused by the generation of piezoelectric potential in PVDF and the internal piezoelectric field limit recombination and accelerate the migration of free charge carriers. As a result, more free charge carriers reaching the surface can participate in the piezocatalysis process and produce more radicals. This can explain the high piezocatalytic activity of composite membranes compared to pure PVDF. The schematic process of piezocatalysis using α-Fe_2_O_3_/PVDF composite is shown in Figure 8.

Under the influence of ultrasound (US) in the membrane, free carriers are formed (reaction (9)). Reaction (9) summarizes several simultaneously occurring processes, which cannot be distinguished. Firstly, under the influence of ultrasonic cavitation, which leads to the generation of high-power acoustic shock waves, PVDF deforms, generating piezoelectric potential and exciting carriers (electrons and holes) at the edges. Secondly, the cavitation-induced collapse of microbubbles, sonoluminescence, and high-temperature lead to the generation of free carriers in α-Fe_2_O_3_.

α-Fe_2_O_3_/PVDF + US → e^−^ + h^+^ + E_F_ (9)

The formed electrons and holes react with dissolved molecules of oxygen and water, forming radicals ^•^O_2_^−^ and ^•^OH, respectively (reactions (10) and (11))

e^−^ + O_2_ → ^•^O_2_^−^ (10)

h^+^ + OH^−^ → ^•^OH (11)

The experiment on radical quenching (Figure 7c) shows that reaction (10) contributes less to the decomposition process. Over time, under the influence of the active forms of oxygen, which have strong oxidizing properties, RhB dye molecules gradually began to decompose (reaction (12)).

^•^OH/^•^O_2_^−^ + RhB **→** Products (12)

**Figure 9:**
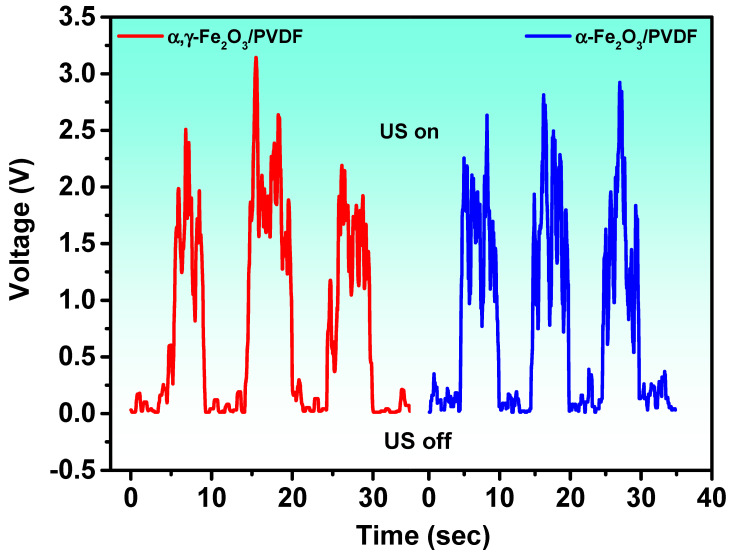
Open-circuit voltage of α-Fe_2_O_3_/PVDF and α,γ-Fe_2_O_3_/PVDF upon switching on and off the US.

To experimentally confirm the generation of piezopotential under conditions where piezocatalysis was carried out, piezoelectric nanogenerators (PNG) were fabricated from α-Fe_2_O_3_/PVDF and α,γ-Fe_2_O_3_/PVDF composite membranes. The measurement results are presented in Figure 9.

Mechanical stimulation of the PNG was performed using a 120 W, 40 kHz US bath, also used for catalytic experiments. The duration and delay between stimulations were 5 s. The generated open-circuit voltage reached an average of about 2.5 V. Under the influence of ultrasound, mechanical stress arises in the composite, creating an internal electric field that promotes effective charge separation.

The long-term stability of the composite membrane, both mechanically and catalytically, is a very important parameter. Therefore, a test on the cycling of the α-Fe_2_O_3_/PVDF membrane during piezocatalytic degradation of RB was conducted. The data are presented in the Supplementary File in Figure S3 [20,23,24,31,32,33,34,35]. It can be seen that the membrane exhibits stable catalytic activity after three cycles at a level of 90%. SEM images of the membrane surface before and after three cycles of piezocatalysis are shown in Figure S4. As can be seen, the membrane is undamaged and defect-free after cycling. Compared to the original membrane, there are no large agglomerates of α-Fe_2_O_3_ after catalysis. Apparently, large surface-bound nanoparticles weakly attached to the matrix during catalysis are carried away into the solution. However, this does not affect the catalytic activity. FTIR spectra before and after three cycles of catalysis were also analyzed (Figure S5), which showed that there were almost no changes except for the appearance of minor noise in the range of 1500–1700 cm^−1^ and 2400 cm^−1^. This may be related to residual Rhodamine B after its piezocatalytic decomposition.

A comparative analysis with composite polymer piezocatalysts reported in the literature in recent years is presented in Table S1 in the Supplementary File [20,23,24,31,32,33,34,35]. We attempted to provide detailed information on all important experimental parameters described in the articles. Based on the provided data, it is obvious that the direct comparison of different catalysts based on rate constant and degradation percentage is incorrect due to the non-standardized procedure of piezocatalytic experiments. Among the important experimental parameters, the following can be noted: percentage of nanoparticle loading in the polymer matrix, thickness of the obtained membrane, sizes and mass of catalyst in the experiment, ultrasound source (bath or probe, power, frequency), reactor sizes and shapes, reactor material, solution volume, etc. Considering all this, it can be concluded that the catalytic activity of our samples is comparable to the state-of-the-art works in this area of research.

## 4. Conclusions

The paper presents the results of the synthesis of porous membranes based on pure PVDF and PVDF doped with α-Fe_2_O_3_ and α,γ-Fe_2_O_3_ nanoparticles. The nanoparticles were obtained by the combustion method. It is shown that doping with phase-pure hematite or mixed-phase hematite/maghemite does not lead to noticeable differences in the structure and catalytic activity of the composite membranes. The samples were investigated using XRD, FTIR, SEM, DRS, PL, and fluorescence microscopy. The combined NIPS/TIPS doctor Blade method of membrane synthesis shows the possibility of achieving a maximum yield of the electroactive phase for pure PVDF and doped membranes. In this case, the proportion of the polar electroactive phase is above 95%. It is shown that nanoparticle doping allows for controlling the pore sizes of the membranes from submicron to subnanometer limits. The membranes exhibit high piezocatalytic activity in the degradation process of Rhodamine B, with the reaction rate using composite membranes being three times higher than that of sonolysis and 1.9 times higher than that of pure PVDF. By using traps and probe labels, it is shown that the main contribution to the degradation process is made by generated radicals ^•^OH and ^•^O_2_^−^. It is shown that the catalyst in piezocatalysis conditions generates a piezopotential of up to 2.5 V.

## Data Availability

Data can be supplied upon request.

## Supplementary Materials

The supporting information can be downloaded at https://www.mdpi.com/article/10.3390/molecules28196932/s1, Figure S1: EDX spectra of samples; Figure S2: XRD patterns of α-Fe_2_O_3_ and α,γ-Fe_2_O_3_ with standards; Figure S3: Cycling tests of the α-Fe_2_O_3_/PVDF membrane in the degradation of RhB; Figure S4: SEM images of α-Fe_2_O_3_/PVDF before and after 3 cycles piezocatalytic experiment and selected area EDX spectra; Figure S5: FTIR spectra of α-Fe_2_O_3_/PVDF before and after 3 cycles piezocatalytic experiment; Table S1: Previously reported work and its comparison with our present work in field Piezocatalytic properties of PVDF based composites.
