# Supplementary material for: Combination NIPS/TIPS Synthesis of α-Fe2O3 and α/γ-Fe2O3 Doped PVDF Composite for Efficient Piezocatalytic Degradation of Rhodamine B
Source: Molecules. 2023 Oct 4;28(19):6932. doi: 10.3390/molecules28196932 (PMC10574218; doi:10.3390/molecules28196932)
Supplement: Supplementary file 1 [file molecules-28-06932-s001.zip › molecules-2647747-supplementary.pdf]

*Article*

# Combination NIPS/TIPS Synthesis of $\alpha$-$Fe_2O_3$ and $\alpha/\gamma$-$Fe_2O_3$ doped PVDF Composite for Efficient Piezocatalytic Degradation of Rhodamine B

**A.G. Magomedova [1], A.A. Rabadanova [1], A.O. Shuaibov [1], D.A. Selimov [1], D.S. Sobola [2,*], K. Rabadanov [2], K.M. Giraev [1] and F.F. Orudzhev [1,3,*]**

[1] Smart Materials laboratory, Department of Inorganic Chemistry and Chemical Ecology, Dagestan State University, St. M. Gadjieva 43-a, Dagestan Republic, 367015 Makhachkala, Russia
[2] Department of Physics, Faculty of Electrical Engineering and Communication, Brno University of Technology, Technicka 10, 616 00 Brno, Czech Republic
[3] Amirkhanov Institute of Physics of Dagestan Federal Research Center, Russian Academy of Sciences, 367003 Makhachkala, Russia
* Correspondence: farid-stkha@mail.ru

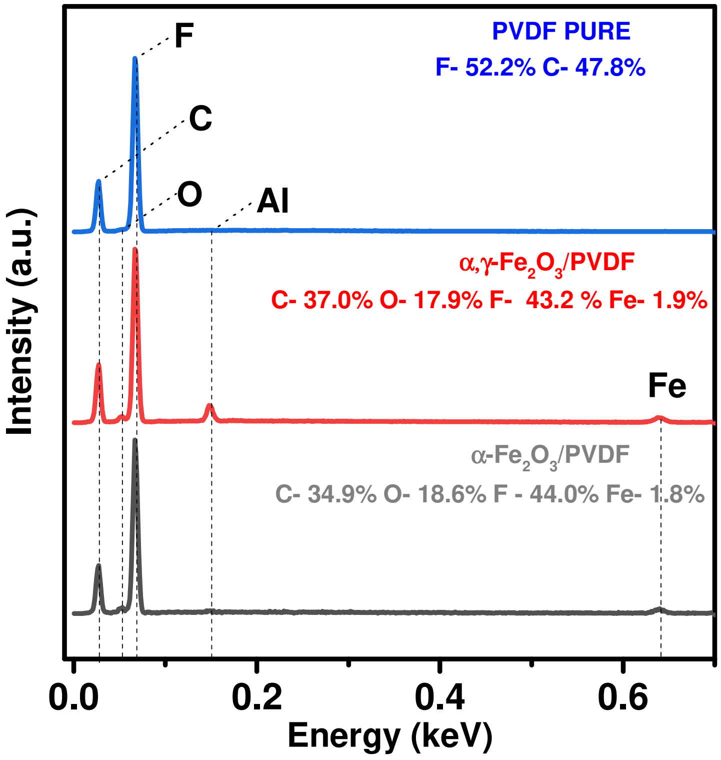


Figure S1. EDX spectra of samples

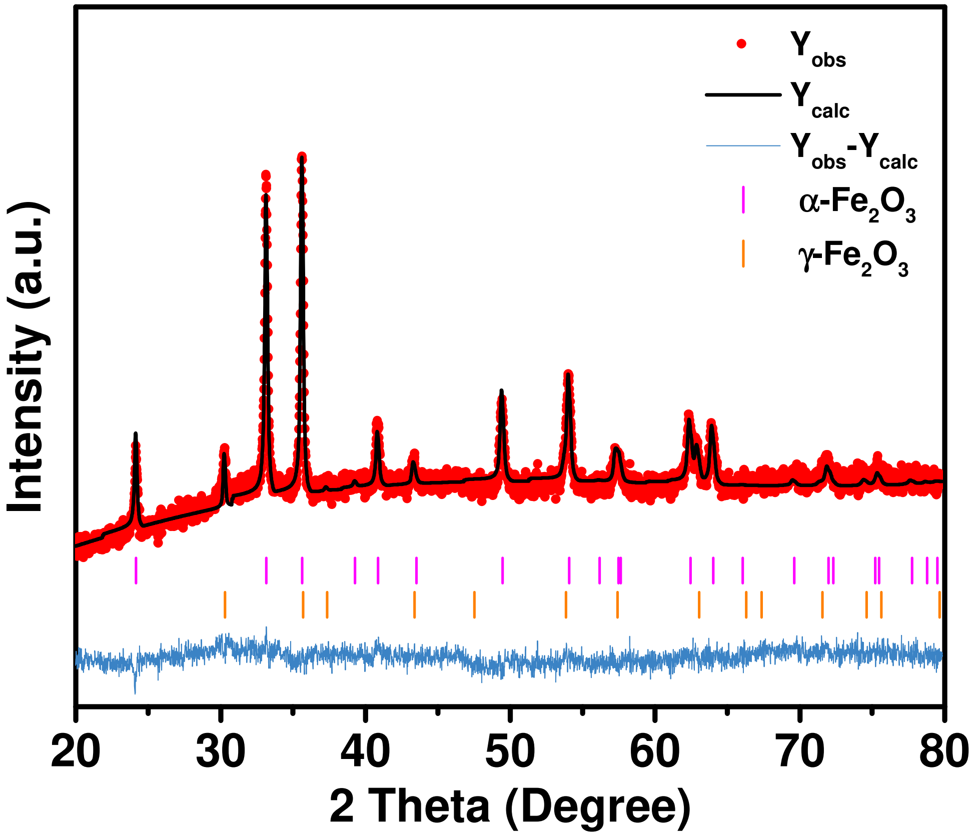


**Figure S2.** XRD patterns of $\alpha$-Fe2O3 and $\alpha$,g-Fe2O3 with standarts.

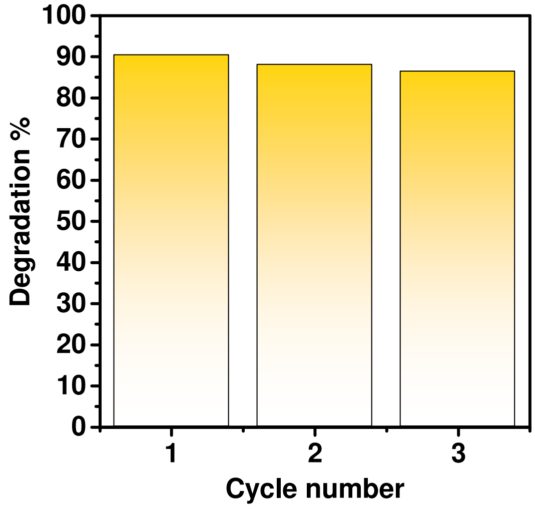


**Figure S3.** Cycling tests of the $\alpha$-Fe2O3/PVDF membrane in the degradation of RhB.

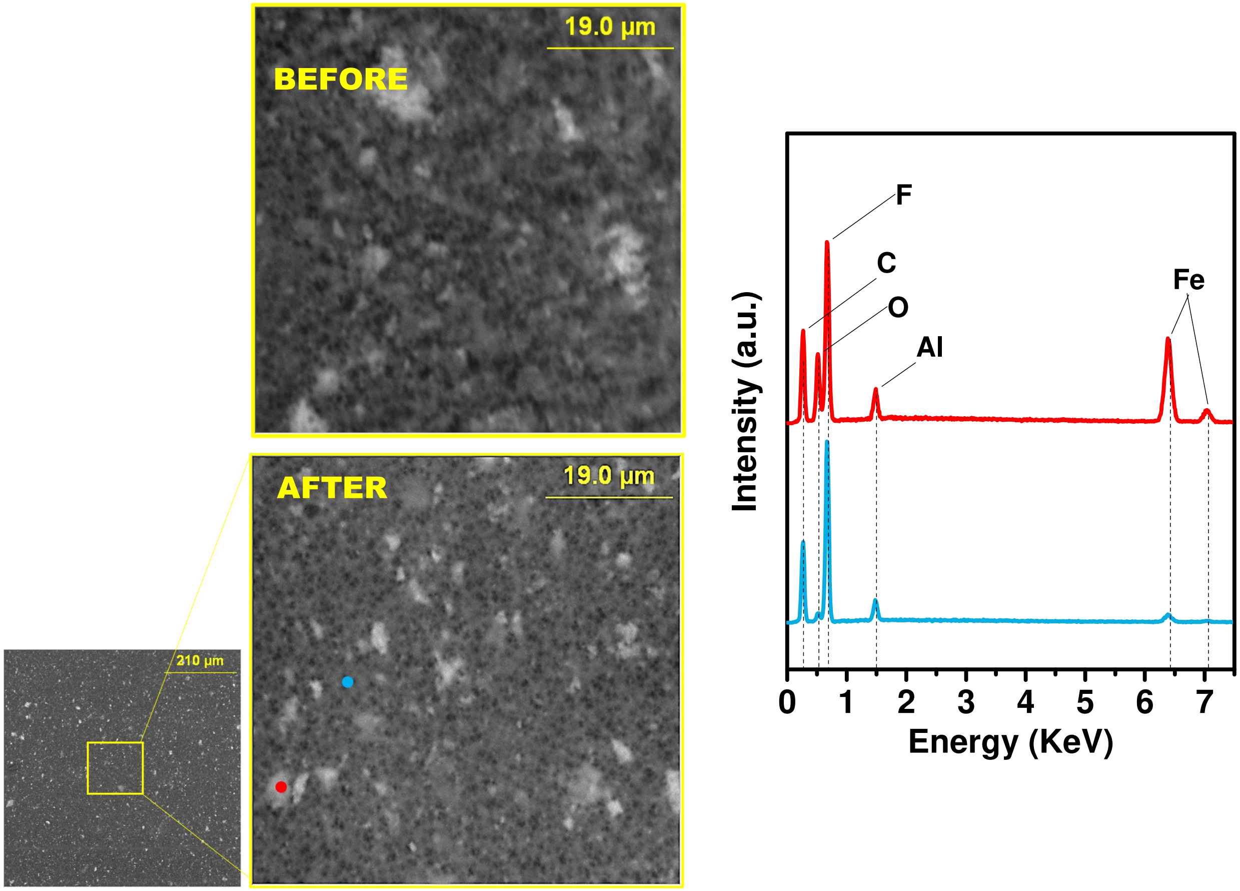


**Figure S4**. SEM images of $\alpha$-$Fe_2O_3$/PVDF before and after 3 cycles piezocatalytic experiment and selected area EDX spectra

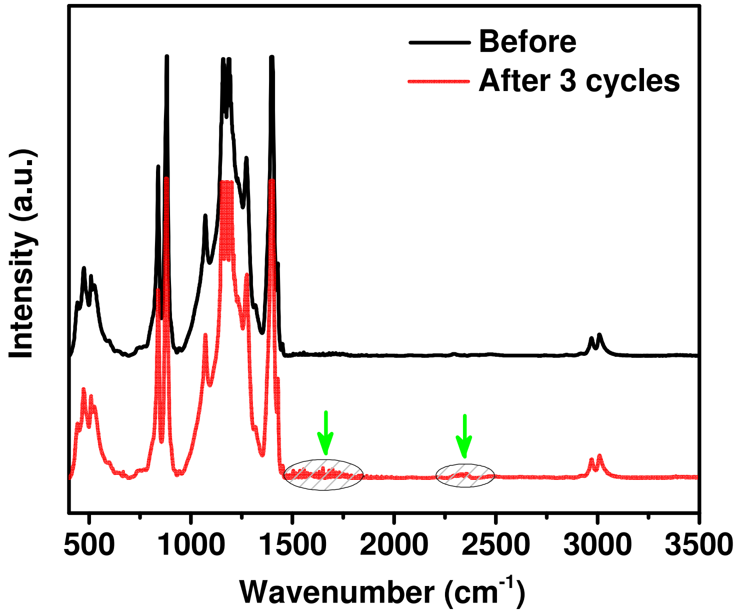


**Figure S5.** FTIR spectra of $\alpha$-$Fe_2O_3$/PVDF before and after 3 cycles piezocatalytic experiment.

**Table. S1. Previously reported work and its comparison with our present work in field Piezocatalytic properties of PVDF based composites.**

| Materials | Pollutants | Time (min) | Mechanical source | Degradation, % | Rate constant, $min^{-1}$ | References |
|---|---|---|---|---|---|---|
| E-$MoS_2$/PVDF EFMs<br>10 wt %<br><br>100 mg | OTC<br>C= 20 mg/L<br>V= 100 mL | 24 | US<br>v= 20 kHz | 93.05 | 0.09124 | 1 |
| Ag@$LiNbO_3$/PVDF<br>5 wt %<br>2.5 cm Diameter | Rhodamine B<br><br>C= 5 mg/L<br>V= 10 mL | 120 | Ultrasonicator<br>W= 70 W<br>v= 40 kHz | ~80 | - | 2 |
| CBO/PVDF<br>(1:1 mass)<br>? | Rhodamine B<br><br>C=10 mg·$L^{-1}$<br>V=? | 10 | Ultrasonic instrument<br><br>W = 100 W | 99.9 | - | 3 |
| $MoS_2$-PVDF<br>10 wt%<br>2×2 $cm^2$<br>thickness 50 μm | Rhodamine B<br><br>C= 10 ppm<br>V= 10 mL | 20 | RS Pro Ultrasonic Cleaner<br><br>W=100 W | >90 | 0.21 | 4 |
| PVDF/$ZnSnO_3$/$MoS_2$<br>20 wt %<br>? | MB<br><br>C= 5 mg/L<br>V=? | 4 | Ultrasonicator<br><br>W= 50 W<br>v= 40 kHz | 100 | - | 5 |
| BTO-PDMS<br><br>(25 wt %)<br><br>? | Rhodamine B<br><br>C= 5 mg/L<br>V= 40 mL | 120 | Ultrasonic machine<br><br>W= 400W<br>v= 40 kHz | ~94 | 0.02254 | 6 |
| CNT/PVDF<br>(SCP, 0.015 g) | Rhodamine B<br>C= 5 mg/L<br>V= 15 mL | 120 | US<br><br>W= 240 W | ≥ 95 | - | 7 |
| $Bi_2ZnB_2O_7$ - Polyacrylonitrile (BBZO-PAN) | MB<br>C= 5 mg/L<br>V= 10 mL | 180 | Ultrasonicator (Labman)<br><br>W=150 W<br>v= 40 kHz | 37 | 2.1*$10^{-3}$ | 8 |

| | | | | | | |
|---|---|---|---|---|---|---|
| $1.5\times1.5\ cm^2$ | | | | | | |
| α-$Fe_2O_3$/PVDF (2wt%) 3x1 $cm^2$ thickness ~110 μm | Rhodamine B (RhB) C= 8 mg/L V= 20 mL | 60 | US bath W=120 W 40 KHz | 90 | 0.036 | This work |


1. Ma, W., Yao, B., Zhang, W., He, Y., Yu, Y., & Niu, J. (2021). Fabrication of PVDF-based piezocatalytic active membrane with enhanced oxytetracycline degradation efficiency through embedding few-layer E-MoS2 nanosheets. *Chemical Engineering Journal*, *415*, 129000.
2. Singh, G., Sharma, M., & Vaish, R. (2021). Flexible Ag@ LiNbO3/PVDF composite film for piezocatalytic dye/pharmaceutical degradation and bacterial disinfection. *ACS Applied Materials & Interfaces*, *13*(19), 22914-22925.
3. Liao, X., Chen, X., Tang, Y., Zhu, M., Xie, H., Xin, Y., ... & Fan, X. (2022). Enhanced Piezocatalytic Reactive Oxygen Species Production Activity and Recyclability of the Dual Piezoelectric Cu3B2O6/PVDF Composite Membrane. *ACS Applied Materials & Interfaces*, *15*(1), 1286-1295.
4. Bagchi, B., Hoque, N. A., Janowicz, N., Das, S., & Tiwari, M. K. (2020). Re-usable self-poled piezoelectric/piezocatalytic films with exceptional energy harvesting and water remediation capability. *Nano Energy*, *78*, 105339.
5. Veeralingam, S., & Badhulika, S. (2023). Rapid Degradation of Organic Dyes via Ultrasound Triggered Piezo-Catalysis Using PVDF/ZnSnO3/MoS2 Nanocomposite. *ACS Applied Nano Materials*.
6. Qian, W., Zhao, K., Zhang, D., Bowen, C. R., Wang, Y., & Yang, Y. (2019). Piezoelectric material-polymer composite porous foam for efficient dye degradation via the piezo-catalytic effect. *ACS applied materials & interfaces*, *11*(31), 27862-27869.
7. Liu, C., Xu, H., Huo, B., Wang, J., Wang, Z., Chen, X., ... & Wang, Y. (2023). Research progress of PVDF based piezoelectric polymer composites in water pollution remediation. *Journal of Water Process Engineering*, *55*, 104181.
8. Porwal, C., Verma, S., Chauhan, V. S., & Vaish, R. (2023). Bismuth zinc borate-Polyacrylonitrile nanofibers for photo-piezocatalysis. *Journal of Industrial and Engineering Chemistry*, *124*, 358-367.
